# The maturation of speech structure in psychosis is resistant to formal education

**DOI:** 10.1038/s41537-018-0067-3

**Published:** 2018-12-07

**Authors:** Natália Bezerra Mota, Mariano Sigman, Guillermo Cecchi, Mauro Copelli, Sidarta Ribeiro

**Affiliations:** 1 Instituto do Cérebro, Universidade Federal do Rio Grande do Norte, Natal, Brazil; 2 Departamento de Física, Universidade Federal de Pernambuco, Recife, Brazil; 3 Laboratorio de Neurociencia, Universidad Torcuato Di Tella, Buenos Aires, Argentina; 4 CONICET (Consejo Nacional de Investigaciones Científicas y Técnicas), Buenos Aires, Argentina; 5 Facultad de Lenguas y Educación, Universidad Nebrija, Madrid, Spain; 6 Computational Biology Center – Neuroscience, IBM T.J. Watson Research Center, Yorktown Heights, USA

## Abstract

Discourse varies widely with age, level of education, and psychiatric state. Word graphs have been recently shown to provide behavioral markers of formal thought disorders in psychosis (e.g., disorganized flow of ideas) and to track literacy acquisition in children with typical development. Here we report that a graph-theoretical computational analysis of verbal reports from subjects spanning 6 decades of age and 2 decades of education reveals asymptotic changes over time that depend more on education than age. In typical subjects, short-range recurrence and lexical diversity stabilize after elementary school, whereas graph size and long-range recurrence only steady after high school. Short-range recurrence decreases towards random levels, while lexical diversity, long-range recurrence, and graph size increase away from near-randomness towards a plateau in educated adults. Subjects with psychosis do not show similar dynamics, presenting at adulthood a children-like discourse structure. Typical subjects increase the range of word recurrence over school years, but the same feature in subjects with psychosis resists education.

## Introduction

Literacy shapes the gradual maturation of discourse. At the individual level, language begins to be learned within weeks of birth if not earlier^1,2^ but its full development takes many years of formal and informal education.^3,4^ Schools are organizations specialized in using the scaffolding of biological maturation to train declarative and procedural skills such as reading and writing, firmly grounded on the progressive expansion of memory capacity and retrieval, coordination, brain area recycling, and symbolic repertoire.^5–8^ While phonological perception and production are typically mastered within the initial years of life, vocabulary, syntax, and grammar continue to mature into high school through a combination of cognitive development and education that is accelerated by alphabetization but undergoes an extended period of subsequent refinement.^4,9,10^

In patients with schizophrenia or bipolar disorder type 1, however, discourse often deteriorates instead of improving, despite schooling and in parallel with the first surfacing of psychotic symptoms.^11,12^ A prospective longitudinal cohort followed from birth until 20 years old of all individuals born between 1991 to 1992 in Avon, England, showed slower cognitive development and increasing deficits since childhood in individuals that at 20 years old had presented a psychotic disorder.^13^ These mental perturbations usually appear between adolescence and early adulthood and progressively impact social behavior and language use.^14–16^ Psychotic discourse is characterized by comparatively reduced vocabulary, short-range repetitions of word sequences, a reduction in long-range themes, and a decrease in the global extent of the word network employed.^11,14,15^

In recent years, we have shown that graph analysis from word trajectories (representing each word as a node and the sequence of words by directed edges) characterizes formal thought disorder in psychosis. The disorganized or overly concrete discourse detected by psychiatric evaluation as a poor and repetitive word trajectory is represented as a graph with smaller long-range recurrence.^17–19^ These graph attributes can successfully predict the Schizophrenia diagnosis in chronic and recent-onset psychosis, as well as negative symptoms that impact social behavior.^17–19^

Does psychosis represent the failure of mental maturation? Could the disorganization of language that results from psychosis represent a developmental impairment of discourse structure? With proper metrics to establish the distance between typical and atypical adults with psychotic symptoms—as proxies of organized and disorganized discourse—we aimed to verify whether verbal reports from typically-developing children move along this dimension as they mature.

**Fig. 1:**
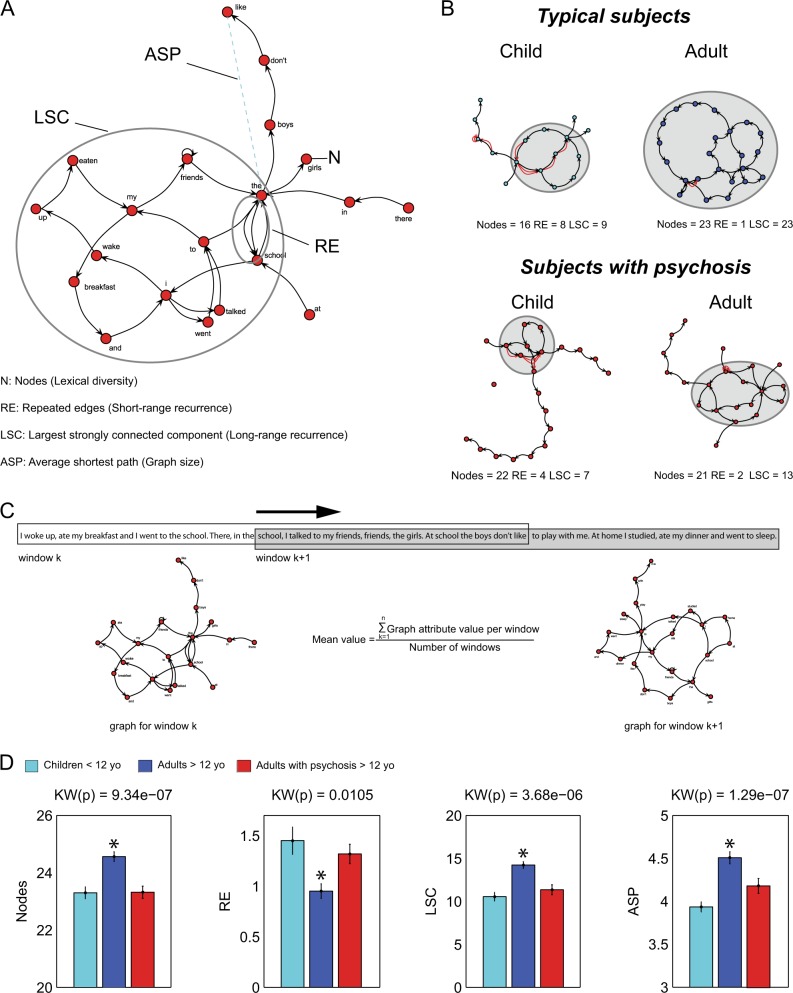
Verbal reports from typical children and psychotic adults are structurally similar and both are different from typical adults. **a** The graph attributes investigated comprised of lexical diversity (N), long-range recurrence (LSC), short-range recurrence (RE), and graph size (ASP).^17,18^ Red circles indicate nodes, black arrows indicate edges. **b** Representative examples of graphs from typical and psychotic subjects, as children or adults. Light grey perimeters indicate LSC. **c** Moving windows (length = 30 words, 50% overlap) were used to calculate mean values per graph for the different attributes. **d** Graph attributes from psychotic subjects are not significantly different from those of typical children (Table 2). KW(p) for Kruskal–Wallis *p* value is shown, and post hoc statistical significance was assessed by the Wilcoxon rank sum test (two-tailed); * indicates significant differences (Bonferroni correction for 4 comparisons, *α* = 0.0125, *p* values in Table 2). Sample sizes: Typical children < 12 yo (years old) (*N* = 80), typical adults > 12 yo (*N* = 55), adults subjects with psychosis > 12 yo (*N* = 63)

To explore this hypothesis, we investigated speech structure using non-semantic graph analysis of 200 interview transcripts (recorded from 135 typical subjects and 65 patients with psychotic symptoms, ages 2 to 58 years old; Table 1). Based on previous findings, we focused here on the following graph attributes: number of nodes (N), which accounts for lexical diversity, repeated edges (RE) and the largest strongly connected component (LSC), which respectively measure short-range and long-range recurrence, as well as average shortest path (ASP), a measure of the graph size (Fig. 1A; see Methods).

**Table 1:** Demographic and psychiatric characteristics of cohort of typical and non-typical (psychotic) subjects

Demographic characteristics	Psychosis	Control
Non-adults	17	93
Adults	48	42
Age	29.51 ± 13.36	14.92 ± 11.61
Male	72% (*N* = 47)	49% (*N* = 66)
Female	28% (*N* = 18)	51% (*N* = 69)
Years of education	7.42 ± 4.61	6.22 ± 6.37

Psychiatric assessment	Schizophrenia	Bipolar
Number of individuals	36 (6 females)	29 (12 females)
BPRS	16.81 ± 6.33	15.28 ± 7.06
PANSS	69.69 ± 14.58	62.45 ± 15.46
Disease duration (years)	12.31 ± 12.44	8.28 ± 9.64
AP typical	67%	59%
AP atypical	47%	28%
Mood stabilizer	11%	62%
Antidepressant	3%	21%
Benzodiazepine	22%	21%

Number of adult and non-adult individuals in each sample (adult considered as equal or above 18 years old). Mean and standard deviation for age in years, sex and years of education. The psychiatric assessment shows number of individuals for each diagnosis (Schizophrenia or Bipolar Disorder), number of females in each diagnostic group, mean and standard deviation for psychometric scale (severity of general and psychotic symptomatology), and disease duration in years, and medication used (in percentage of patients using a medication class in each diagnostic group). Note that there is an imbalance regarding sex distribution in psychotic sample (specifically subjects with schizophrenia diagnosis). Another important note is that there are many more children in the Control sample, due to the difficulties of diagnosing psychosis during childhood. This difference impacts the distributions of age and years of education. Abbreviations: Brief Psychiatric Rating Scale (BPRS), Positive and Negative Syndrome Scale (PANSS), anti-psychotic (AP)

Our previous results lead us to predict that as typical subjects age and undergo schooling, their memory reports should progressively increase in lexical (node) diversity (N), long-range recurrence (LSC), and graph size (ASP). On the other hand, short-range recurrence (repeated edges—RE) should gradually decrease (Fig. 1A). Reports from psychotic subjects should not show the same dynamics, i.e., we hypothesize that the same four graph attributes will be less correlated with age or years of education, remaining similar to those of typical children’s reports.

## Results

**Fig. 2:**
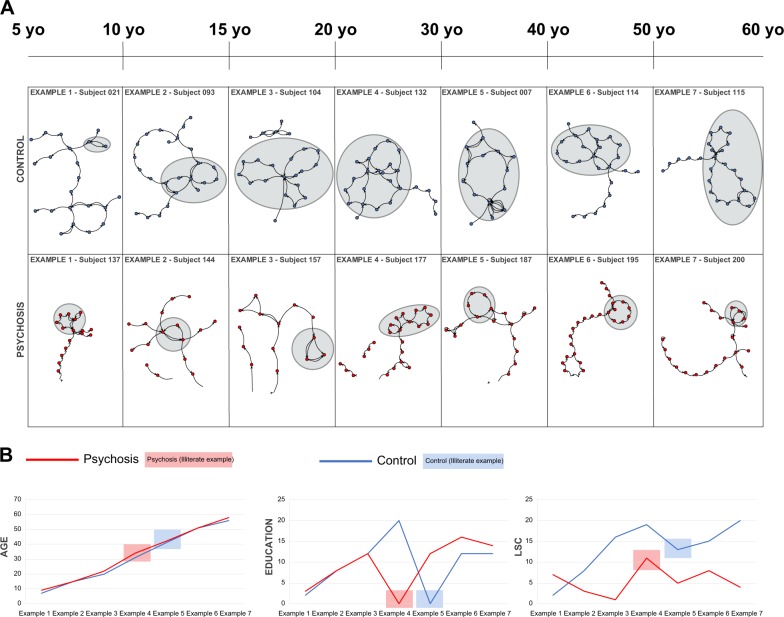
Qualitative representation of LSC across subjects spanning six decades of age and 2 decades of education. **a** Representative examples of graphs (initial 30 words) from subjects in the control subjects (blue dots) and patients with psychosis (red dots). Ellipses in grey denote LSC. **b** The LSC dynamic range for the examples shown above is twice as large for the control subjects than for the patients with psychosis. Data from two illiterate adults with no formal education (highlighted in red and blue) are shown. Note that the lack of education seems to greatly impact LSC among control subjects, but not among patients with psychosis

We analyzed the graph representation of memory reports from 200 typically-developing individuals and 65 individuals with psychotic symptoms (Fig. 1A). To account for non-pathological verbosity differences across subjects, graph analysis used a sliding window with a fixed length of 30 words, with 50% overlap between consecutive windows (Fig. 1C). The four graph attributes differed as predicted between typical subjects below and above 12 years of age, indicating a change towards more organized discourse (Fig. 1D, light and dark blue columns; representative examples in Fig. 1b and Fig. 2, statistics in Table 2). Before the age of 12 years the diagnosis of psychosis is very rare, and for this reason we used that age cutoff for our initial analyses.^20–22^ As expected, psychotic subjects produced reports that structurally resembled the disorganized pattern seen in typical subjects with less than 12 years of age (illustrative examples in Fig. 1b and Fig. 2. Figure 1d, light blue and red columns, statistics in Table 2).

**Table 2:** Statistical analysis of graph attribute differences between groups (typical children, adults and psychotic subjects)

	Nodes	RE	LSC	ASP
Statistical analysis (*p* values)				
KS test	**<0.0001**	**<0.0001**	**<0.0001**	**<0.0001**
Levene test	0.0088	<0.0001	0.0010	0.0411
Kruskal–Wallis test (*p* values)				
Psychosis × children × adults	**<0.0001**	**0.0105**	**<0.0001**	**<0.0001**
Wilcoxon Ranksum test (*p* values)				
Children × typical adults	**<0.0001**	**0.0079**	**<0.0001**	**<0.0001**
Psychosis × children	0.6992	0.9077	0.3311	0.0156
Psychosis × typical adults	**<0.0001**	**0.0074**	**0.0002**	**<0.0007**

P values from Kolmogorov-Smirnov (KS), Levene, Kruskal-Wallis and Wilcoxon ranksum tests (corrected for 4 comparisons, α = 0.0125). Statistical significance indicated in bold face

Representative graphs illustrate the marked structural differences between typically-developing children and adults, not present in subjects with psychotic symptoms (Fig. 1b and Fig. 2). In support of our hypotheses, three attributes of interest (N, LSC, ASP) showed significant positive Spearman correlations with both age and education in typical subjects (Table 3). Note that this correlation does not assume a linear model. The short-range recurrence attribute RE, which in typically-developing children is negatively correlated with Intelligence Quotient and Theory of Mind scores,^23^ showed a significant negative correlation with education but not with age in typical subjects (Table 3). In striking agreement with our prediction that psychotic language remains in a disorganized stage, none of the graph attributes changed significantly either with age or with education among subjects with psychosis (Table 3). A multiple linear regression confirmed the predominance of education over age in typical subjects (Table 3). Furthermore, the correlations with age lost significance when corrected for education (all *p* values > 0.05, Table 3), while the correlations with education remained significant when corrected for age (Table 3). This means that the relationship with age is dependent on education, but the relationship with education is age-independent.

**Table 3:** Correlations and Goodness of fit parameters

Age	Spearman correlation	Nodes	RE	LSC	ASP
Typical	Rho/*p*	**0.36/<0.0001**	−0.22/0.0118	**0.40/<0.0001**	**0.41/<0.0001**
Adjusted for Education	Rho/*p*	−0.09/0.3121	0.10/0.2295	0.06/0.4976	−0.02/0.8381
Psychosis	Rho/*p*	−0.02/0.8919	−0.04/0.7744	0.17/0.1806	0.06/0.6178

Education	Spearman correlation	Nodes	RE	LSC	ASP
Typical	Rho/*p*	**0.49/<0.0001**	−**0.33/0.0001**	**0.45/<0.0001**	**0.51/<0.0001**
Adjusted for age	Rho/*p*	**0.36/<0.0001**	−**0.28/0.0013**	**0.23/0.0064**	**0.33/0.0001**
Psychosis	Rho/*p*	0.06/0.6578	−0.01/0.9253	0.19/0.1294	0.17/0.1750
	**Multiple linear combination**	**Nodes**	**RE**	**LSC**	**ASP**
	*R*²	0.16	0.09	0.23	0.26
	*p*	**<0.0001**	**0.0025**	**<0.0001**	**<0.0001**
	Coef AGE	−0.0067	0.0023	0.0578	0.0050
	Coef EDU	0.1195	−0.0500	0.2353	0.0394
	Coef EDU—Coef AGE	0.1128	0.0478	0.1776	0.0344

Fit for years of Education/Age	Goodness of fit (education/age)	Nodes	RE	LSC	ASP
Control	R Square	0.85/0.82	0.95/0.95	0.83/0.80	0.52/0.44
	SSE	7.81/9.50	0.63/0.66	45.58/54.71	0.36/0.40
	RMSE	0.53/0.57	0.15/0.15	1.28/1.40	0.11/0.42
	f∞.	24.56/24.59	1.07/1.02	18.68/16.09	4.94/0.12
	*T*	0.63/1.95	0.28/1.40	13.34/11.55	11.06/4.67
	f0	19.43/1.00	4.33/29.00	8.32/5.44	3.85/10.16
	|f∞- f0|	5.13/23.59	3.26/−27.98	10.36/10.65	1.08/−10.04
Psychosis	*R* Square	0.01/0.02	0.01/0.12	0.42/0.15	0.05/0.20
	SSE	9.16/9.07	1.96/1.75	137.30/200.97	1.33/0.18
	RMSE	0.53/0.52	0.24/0.23	2.04/2.47	0.20/1.11
	f∞.	22.53/23.14	1.55/1.40	18.84/12.12	4.43/0.18
		29.99/8.20	1.12/4.32	14.94/8.81	3.71/4.49
	f0	23.48/25.12	0.00/11.93	6.69/1.00	3.59/6.83
	|f∞- f0|	0.95/−1.97	1.55/−10.52	12.15/11.12	0.85/−6.65

Spearman, partial Spearman and multiple linear correlations with significant p values indicated in bold face. For Spearman correlations, Bonferroni correction for 8 comparisons (2 groups * 4 attributes), alpha = 0.0063. For partial Spearman correlations applied only to the control group, Bonferroni correction for 4 comparisons, alpha = 0.0125. Coef stands for coefficient. Goodness of fit and parameters of exponential model for Age and Education

**Fig. 3:**
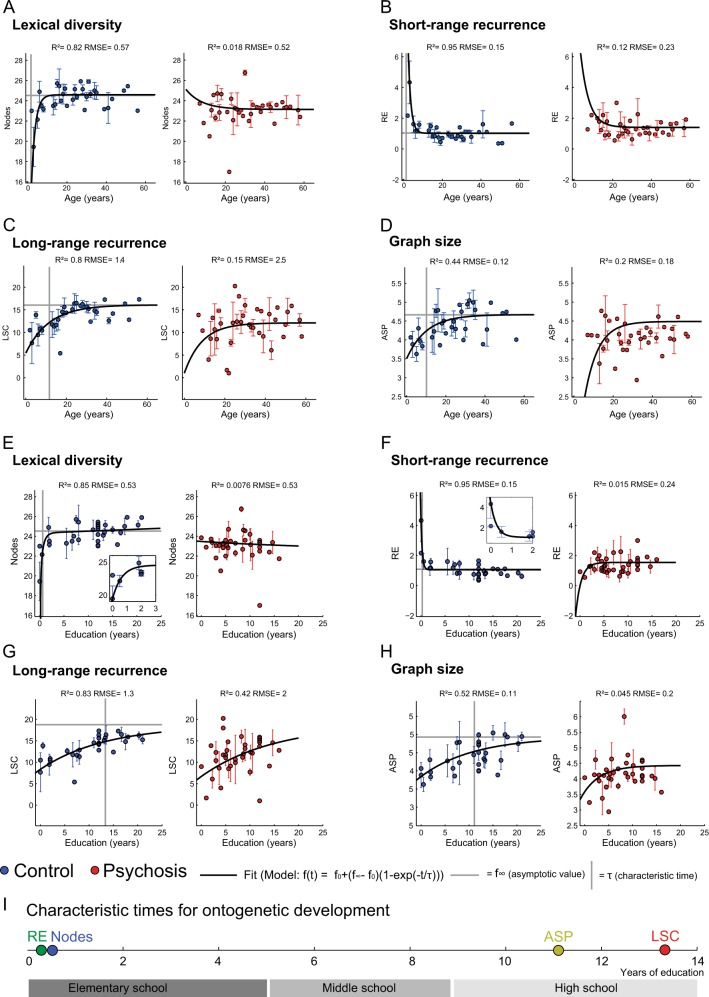
The structure of memory reports matures with years of age and education in typical subjects, but not in psychotic patients. (A) Lexical diversity as a function of years of age (yA) for typical (*N* = 135) and psychotic (*N* = 65) subjects. Similar plots for **b** short-range recurrence, **c** long-range recurrence, and **d** graph size. e Lexical diversity as a function of years of education (yE) for typical (*N* = 135) and psychotic (*N* = 65) subjects. Similar plots for **f** short-range recurrence, **g** long-range recurrence, and **h** graph size. For significant Spearman correlations, characteristic years of age or education (Ƭ) and asymptotic values (*f*_*∞*_) indicated by vertical and horizontal dashed lines, respectively. *R*² and Root-mean-square error (RMSE) indicated on top. For information about the model and parameters used, see Methods and Table 5. For data on Spearman correlations, partial Spearman correlation and multiple linear combinations between education and age, see Table 3. Goodness of fit in Table 3, randomization analysis in Suppl. Figure S1. (I) Characteristic times (Ƭ) showed on temporal order of maturation for specific graph attributes, indicated by colored circles (details in Methods)

To further characterize these changes, graph attribute values were binned in years of age and education and fit with an exponential model weighted for the standard error of the mean. Graph attributes obtained from typical subjects adjusted very well to the model (Fig. 3a–d for age and Fig. 3e–h for education, blue panels, statistics in Table 3), with an age and education-related exponentially saturating increase in lexical diversity (Fig. 3a for age and 3e for education), and a corresponding decrease in short-range recurrence (Fig. 3b for age and 3f for education). Long-range recurrence (Fig. 3c for age and 3g for education) and graph size (Fig. 3d for age and 3h for education) showed a much slower saturating increase. In agreement with our hypothesis that the organization of psychotic discourse changes less through years of age or education, the graph parameters obtained from the recordings of psychotic subjects adjusted poorly to the model (Fig.3a–h, red panels, statistics in Table 3). The prediction that |*f*_*∞*_−*f*_*0*_| would be larger in typical subjects than in subjects with psychotic symptoms was confirmed for lexical diversity (N), short-range recurrence (RE) and graph size (ASP), but not for long-range recurrence (LSC) (Table 3).

In typical subjects, word repetitions (RE) decreased exponentially within the first year of formal education, in parallel with a saturating increase in lexical diversity (N). Graph size (ASP) also increased, but with much slower dynamics that begins to saturate around the beginning of high school. Long-range recurrence (LSC) behaved similarly, with a characteristic time near the end of high school. To further test the null hypothesis of lack of temporal structure in the data, the temporal order (years of age or years of education) of the samples was randomized 1000 times and the graph attributes of this surrogate dataset were compared to real data. Such disruption of temporal order abolished significant Spearman correlations (Suppl. Fig. S1A for age and **C** for education) and greatly reduced the *R*^2^ of the exponential models (Suppl. Fig. S1B for age and **D** for education).

**Fig. 4:**
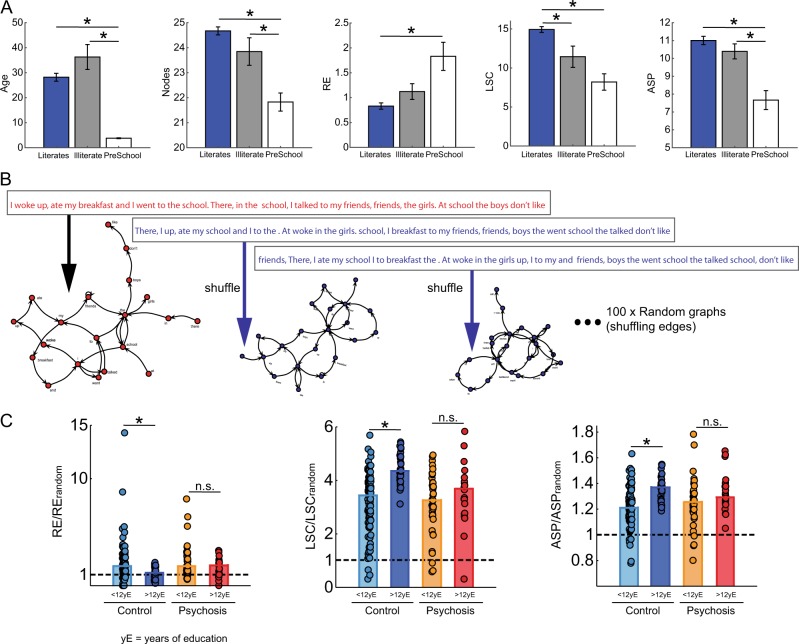
Memory reports from typical illiterate subjects show smaller LSC compared to literate subjects with same age, and psychotic subjects have a near-random structure. **a** Independent sample of illiterate adults (*N* = 14, grey bar), and pre-school children (*N* = 18, white bar) for comparison with a literate adult sample without school dropout (*N* = 41, blue bar). For a comparison between groups with no age difference (literate × illiterate adults, *p* = 0.2267) illiterates produced memory reports with smaller LSC (*p* = 0.0111), but non-significant difference for Nodes (*p* = 0.0927), RE (*p* = 0.0889) or ASP (*p* = 0.1583). For a comparison between groups with no education difference (pre-schooler children × illiterate adults) the pre-school sample produced memory reports with less lexical diversity (*p* = 0.0082), and graph size (*p* = 0.0011), but non-significant differences for RE (*p* = 0.1141), or LSC (*p* = 0.0402). Literate adults differ from pre-school children in all attributes (*N* (*p* < 0.0001), RE (*p* = 0.0016), LSC (*p* < 0.0001), and ASP (*p* < 0.0001)). Bar plots indicate median values and error bars indicate standard error. * For *p* < 0.05 corrected for multiple comparisons, (Wilcoxon rank sum test, two-tailed, Bonferroni correction for four comparisons, *α* = 0.0125). **b** Graph attributes were calculated for each random graph and averaged to compose the denominator of the ratio shown as normalized graph attribute in the next panel. **c** The graph attributes of each individual report were normalized by the corresponding mean random value, and the data were sorted according to more or less than 12 yE (years of Education). Typical subjects showed significant differences between subjects below (<) or above (>) 12yE (*p* for RE = 0.00004, LSC = 1.19e-10, ASP = 8.04e-8), but psychotic subjects did not (*p* for RE = 0.6128, LSC = 0.3712, ASP = 0.1398). *p < 0.05 corrected for multiple comparisons, n.s. for non-significant differences (Wilcoxon rank sum test, two-tailed, Bonferroni correction for six comparisons, *α* = 0.0083)

If education is a major factor to explain the typical development of discourse structure, we expect to find differences between reports from literate and illiterate typical adults of similar age. Since psychosis itself can interfere with education (to the extent that the education lag may indicate the extent of illness), we did not expect the same result in the psychosis group. School drop-outs were more frequently found in the psychotic sample (62% × 28%, *X*² *p* = 0.0002), and education was better correlated with age in the control group than in the psychosis group (control Rho = 0.82, *p* < 0.0001, psychosis Rho = 0.26, *p* = 0.0335). Next, we compared reports from typical adults without any episode of school drop-out (*N* = 41) with reports from an independent sample of illiterate adults (*N* = 14, Fig. 4a). As expected, for subjects of similar ages, literate adults produced reports with significantly larger LSC than illiterate adults (*p* = 0.0111, Bonferroni corrected for four comparisons, Fig. 4a). If we pool the illiterates with subjects with some episode of school drop-out (*N* = 30) and compare them with subjects that never dropped out of school (*N* = 41), the latter produced reports with larger LSC (*p* = 0.0006) and fewer RE (*p* = 0.0063) than the former. There were no significant differences for N or ASP, neither in the control nor in the psychosis group, when adult subjects with and without school drop-out episodes were compared (Psychosis group: *N* = 24 without school dropout and 39 with school dropout, Wilcoxon Ranksum *p* > 0.0125).

To further investigate the roles of education and age, we compared verbal reports of two cohorts that share the lack of education, but very much contrast in age: The fully illiterate adults differed significantly from pre-school children only in lexical diversity and graph size, but not on short or long-range recurrence (RE and LSC respectively, Fig. 4a), while literate adults showed significant differences from preschoolers for all graph attributes considered (Fig. 4a).

If memory reports from subjects with psychotic symptoms are more disorganized than the reports of educated typical adults, it is conceivable that their structure is also closer to that of random graphs.^24^ To gain insight into the structural randomness of our samples, each graph was randomized 100 times by keeping the nodes and shuffling the edges (Fig. 4b). Normalizing each graph attribute by the corresponding mean random graph attribute, LSC and ASP from typical controls with more than 12 years of education (yE) were significantly larger than in controls with less than 12 yE (Fig. 4c). RE showed the opposite profile: Above random in typical controls with less than 12 yE, and near-random in typical controls with more than 12 yE. None of these education-related differences in discourse structure were significant in subjects with psychotic symptoms (Fig. 4c).

Previous studies have detected a significant anti-correlation between LSC (connectedness) and negative symptoms.^18,19,25^ Therefore, an alternative interpretation for the lack of correlation with age or education in the psychosis group is that negative symptoms are better predictors of LSC variance than either age or education. As expected, there was a significant anti-correlation between LSC and the total of PANSS negative subscale (Rho = −0.29, *p* = 0.0183), which remained significant after adjusted by either age (Rho = −0.25, *p* = 0.0423) or education (Rho = −0.26, *p* = 0.0355).

Finally, we considered additional confound factors to explain the structural variance across time. There was no sex-related difference for any graph measures in any group (Table 4), nor were there significant correlations when separately considering subjects with Schizophrenia or Bipolar disorder diagnosis (Table 4). Chlorpromazine equivalent doses were not correlated with graph attributes, and adjustment for medication did not change the correlations between graph attributes and education (Table 4). Family income was the only confound factor that correlated significantly with graph attributes, but this correlation did not remain significant after being adjusted by education. In contrast, the correlation between graph attributes and education remained significant after adjustment for income (Table 4).

**Table 4:** Confound analysis considering sex, family income for both groups, diagnosis, and medication for psychosis group.

Confounds	*N*	RE	LSC	ASP
Sex difference (Wilcoxon Ranksum)—Both groups				
Control (p)	0.7362	0.3391	0.8361	0.7067
Psychosis (p)	0.4546	0.3910	0.8834	0.9942
Income (Spearman correlation with family income)—Both groups				
Control (Rho/p)	**0.23/0.0113**	−0.13/0.1514	**0.26/0.0027**	**0.30/0.0008**
Psychosis (Rho/p)	0.07/0.5614	−0.01/0.9682	0.12/0.3530	0.22/0.0900
Partial correlation (with income adjusted by education)—Control group				
Control (Rho/p)	0.14/0.1222	−0.07/0.4395	0.16/0.0764	0.20/0.0255
Partial correlation (with education adjusted by income)—Control group				
Control (Rho/p)	**0.43/0.0000**	−**0.31/0.0005**	**0.41/0.0000**	**0.45/0.0000**
Diagnosis (Spearman correlations with education)—Psychosis group				
Schizophrenia (Rho/p)	0.11/0.5315	−0.05/0.7690	0.08/0.6358	−0.10/0.5711
Bipolar (Rho/p)	−0.14/0.4638	0.15/0.4336	0.23/0.2362	0.07/0.7373
Medication (Spearman correlation with Chlorpromazine equivalent dose)—Psychosis group				
Psychosis (Rho/p)	0.06/0.6411	−0.14/0.2798	0.03/0.8069	−0.02/0.8723
Partial Correlation (with education adjusted by dose)—Psychosis group				
Psychosis (Rho/p)	0.06/0.6134	−0.03/0.8128	0.20/0.1209	0.17/0.1815

The analysis performed is described on top of each set of results. Spearman correlation with Bonferroni correction for 4 comparisons (4 different attributes, alpha = 0.0125)

## Discussion

Here we present for the first time a non-semantic graph-based description of how schooling may gradually change the way people speak and how psychosis may affect this process. Throughout the school years, verbal discourse becomes less repetitive, richer in vocabulary, and more structured in the long range, so that words recur in a greater number of “word-vicinity” contexts. The benefits of education are lost in subjects with psychotic symptoms, whose verbal production structurally resembles that of children.

The results reveal different scales for the typical maturation of distinct aspects of discourse structure, confirming the expectation of a protracted dynamics of characteristic times, which either precede or coincide with adolescence. That these changes span the entire period of regular schooling points to the importance of high school completion.^26^ Education, which may increase with age but is not reducible to it, shapes the structural modification of discourse from early childhood to adolescence. This process requires time, but developmental time per se does not suffice without education. Also, the results resonate with the notion that adult psychosis reflects childish residues.^27^ This is likely related to developmental limitations in working memory and attention,^28^ which subside with education.^29^ Not surprisingly, limitations also observed in patients with psychotic symptoms.^30^

In literate societies, cultural exposure to written discourse begins early in childhood and extends over life by way of social interactions with literate individuals. Despite this influence, speech structure only begins to mature after alphabetization, as subjects adapt to the standards adopted by literate adults. Subjects with psychosis have difficulties in social interaction, maintaining a non-semantic discourse structure similar to that of children or illiterate adults: Although they have been immersed for a long time in the literate culture, full literacy never developed.^23,31^

Of note, the failure of discourse maturation in psychosis does not mean that typical children have language similar to that of adults with psychosis. By analogy, an adult with dwarfism may have the same height as a child, but typical children do not have dwarfism. It is important to emphasize that long-range recurrence (speech connectedness, LSC) in subjects with psychosis varied not according to age or education, but according to negative symptoms, as previously reported.^18,19,25^ We have recently shown that decreased speech connectedness (LSC) is correlated with a decreased degree of graph centrality in resting state functional MRI data, as well as with a decreased cortical gyrification index, as measured by structural MRI.^25^ Importantly, speech connectedness (LSC) was also correlated with the psychometric evaluation of thought disorder, with processing speed deficits and with the functional outcome of the patients. This highlights the relevance of cognitive deficits to explain the variance of speech structure when a psychotic condition is present.

Another possible interpretation of the results is that a poor and repetitive word trajectory may reflect a more concrete discourse with low syntactic complexity, rather than discourse disorganization.^32^ This is in agreement with the fact that schizophrenia subjects with formal thought disorder speak with more referential anomalies and less syntactic complexity than healthy controls.^33^ Please note that the use of LSC as a proxy of syntactic complexity does not necessarily correspond to its definition in linguistics as the number of embedded clauses or dependents. Future investigation shall elucidate the precise relationship between graph-theoretical and linguistic measures in the discourse of subjects with psychosis, as well as the psychosis-related differences in the use of syntactic vs. semantic words (i.e., functional words such as possessive and relative pronouns, articles, and determiners, versus referential/content words such as nouns).^32^

Additionally, there may be other deficits in related or homologous brain regions that underlie social communication (through prosody and facial expression) that contribute to poor social function, as well as social anxiety and motivational deficits (which may or may not be secondary to communication deficits). In schizophrenia, for example, reading disabilities such as acquired dyslexia are correlated with visual and phonological impairment.^31^ Future studies linking brain function and speech structure shall elucidate these questions.

The characteristic times of graph attributes are summarized in Fig. 3i. Education-related cultural accumulation makes discourse less recursive and more connected. Short-range recurrence and lexical diversity begin to stabilize in the first school year, as expressed in a wider use of an expanding vocabulary and less use of mnemonic resources to organize a speech. This is consistent with evidence that lexical connectivity facilitates language acquisition even in preschool children.^8^ Then, mostly during high school but with large inter-individual variation, graph size and long-range recurrence saturate, and graph attributes evolve towards the typical adult profile. The characteristic times (Ƭ) when graph attributes were modeled according to age (Table 3) provide post-hoc validation of the 12 yo cut-off adopted early on for the analysis.

The data point to a hierarchical development of discourse structure, by which we depart from an initial pattern of fragmented word segments dominated by short-range connections to a learned pattern of globally connected word strings. Importantly, long-range recurrence (LSC), the main graph attribute that differentiates schizophrenia diagnosis^17–19^ had lower *f*_0_ values in the psychotic sample than in the typical sample, while *f*_∞_ values were more similar across groups. Thus, the long-range recurrence deficit in subjects with psychotic symptoms may reflect not a return to an immature pattern, but rather a developmental course that strays from the typical profile from start, as suggested by a prospective longitudinal study.^13^

It is important to consider that the same graph attributes that decline during psychosis in association with cognitive deficits increase over typical development in children learning the alphabet, reading and writing.^23^ This rise correlates with reading performance, IQ and theory of mind,^23^ three important measures of cognitive and social skills required for collective integration. However, the lack of such data for most subjects precluded an analysis of this confound variable.

While the complex discourse structure of typical adults owes more to nurture than to nature, education does not do its work in subjects with psychosis. When cognitive development is impaired by disease, nature trumps nurture. Despite exposure to education, subjects with psychosis retain a linguistic structure akin to that of children’s speech, failing to mature in complexity and remaining closer to a near-random structure. The question of whether psychosis represents a failure of mental development is not new, and the answer remains unclear. Yet, our findings have important implications for early intervention of psychosis in educational settings and hold the promise of improving computational assessment for early intervention. The school environment is strategic for the early identification of risk. A closer look at cognitive development using computational assessments in naturalistic school settings can enable early interventions to mitigate cognitive damages.^13,34,35^

## Methods

### Subjects

The convenience sample was pooled from^18,19,23,36^ plus new samples and comprised clinical oral interviews from 200 individuals (135 without any diagnosis of psychiatric disorder, age of 2 to 56 years old and 65 independently diagnosed by the standard DSM IV ratings SCID^37^ with psychotic symptoms as schizophrenic (S) (*N* = 36) or bipolar type I (B) (*N* = 29), age of 7 to 58 years old, Table 1). Also applied were two standard psychometric scales, the “Positive and Negative Syndrome Scale” (PANSS)^38^ and the “Brief Psychiatric Rating Scale” (BPRS),^39^ and a socioeconomic-clinical questionnaire (with information regarding age, sex, family income, educational level, marital status, disease duration, and onset). Both samples includes subjects that dropped out of school, as is typical of Brazil overall, and of the state of Rio Grande do Norte in particular (respectively, 18.1% and 25.2% distortion between age and educational level.^40^ An independent sample of illiterate adults was also investigated (*N* = 14). The study used data from two protocols approved by the Research Ethics Committee of the Federal University of Rio Grande do Norte (permits #102/06-98244 and #742.116). Signed informed consent was obtained from all participants and also from a legal guardian when necessary, and the study adhered to all relevant ethical regulations. The exclusion criteria were any neurological condition or alcohol/drug abuse. Some controls were medicated for anxiety and depression (Table 1). The analysis of memory reports focused on answers to three open questions, namely requests for reports on one recent dream, on waking activities in the previous day, and about a negative affective image shown for 15 s immediately before the request. The negative image was selected from a widely validated affective images database.^41^ For each subject, the three reports were concatenated and the final text was represented as a word graph (Fig. 1a).

### Non-semantic word graph analysis

The data are fully available at the Suppl. Tables S1. Non-semantic word graph analysis was performed using the software *SpeechGraphs*, which is freely available at http://www.neuro.ufrn.br/softwares/speechgraphs. The representation of the text as a graph consisted in assigning to each word a node and to each sequence of consecutive words a directed edge (Fig. 1a). Lemmatization was not performed because we had previously determined—for the purposes of Schizophrenia diagnosis—that non-semantic word graph analysis yields very similar results for lemmatized^17^ or non-lemmatized^18,19^ data. Average graph attributes were calculated using moving windows of 30 words with 50% of overlap,^18^ i.e., steps of 15 words (Fig. 1c), and calculating graph attributes for each resulting graph. A total of four average graph attributes were calculated for each text file, comprising lexical diversity (nodes = N), short-range recurrence (repeated edges = RE), long-range recurrence (largest strongly connected component = LSC) and graph size (average shortest path = ASP). RE corresponds to the sum of all edges linking the same pair of nodes. LSC corresponds to the number of nodes in the maximal subgraph in which all pairs of nodes are reachable from one another in the directed subgraph (i.e., node *a* reaches node *b*, and vice-versa). ASP corresponds to the average length (number of steps along edges) of the shortest path between pairs of nodes of a network. To estimate randomness levels, each 30-word window was shuffled 100 times so as to keep the same words but change their order (Fig. 4b). This procedure is equivalent to a random permutation of edges.^24^ Graph attributes of randomized word windows were then averaged and used to normalize the original average data. Data analyzed in Excel and Matlab software.

### Exponential model

In order to study the dynamics of graph attributes across different ages or educational levels, the following model was used:

1 $$f\left( t \right) = f_0 + \left( {f_\infty - f_0} \right)\left( {1 - exp\left( { - t/T} \right)} \right)$$

where *f*_∞_ is the maximum asymptotic graph attribute value, *f*_0_ is the initial graph attribute value, t is time, and *T* is characteristic time to reach saturation.

The function is the solution to a linear differential equation of first order:

2 $${\mathrm{df/dt}} = \left( {1{\mathrm{/}}T} \right)\left( {f_\infty - f} \right)\,{\mathrm{with}}\,{\mathrm{initial}}\,{\mathrm{condition}}\,f\left( {t = 0} \right) = f_0$$

The evolution of each attribute was modeled as an exponential fit to represent accelerated initial development followed by a saturation process of slow progress. This fit to exponentials allows us to identify dynamic properties of each attribute. We chose to adjust the data to the simplest possible model, one that only presupposes linear dynamics that converges to a stable fixed point. This provides useful parameters to interpret the data and sets the stage for specific predictions:

The saturation onset should either precede or coincide with adolescence when it becomes possible for the first time to clinically identify the losses produced by psychosis.^42^ Furthermore, if discourse in typical children shifts through development from disorganized to organized, but remains largely disorganized in psychotic subjects, we expect initial and asymptotic graph attribute values to be quite different in the former, but not in the latter, i.e., |*f*_∞_*−f*_0_| should be greater in typical subjects than in psychotic patients. Furthermore, typical subjects should show *f*_∞_ > *f*_0_ for N, ASP, and LSC, but *f*_0_ *>* *f*_∞_ for RE. On the model, we used as input data the average graph attribute from all individuals with the same age and weighted the model for the standard error of the mean. To better adjust the fit, we considered lower and upper points to each coefficient, according to the maximum and minimum value expected for each graph attribute and for time (years of age and education), as detailed in Table 5. In order to further evaluate the model’s goodness of fit, we shuffled the temporal variable 1000 times, using years of education (Suppl. Fig. S1).

**Table 5:** Parameters and rationales for the exponential model

Coefficient	Rationale for lower point	Rationale for upper point	Start-point
*f*_*∞*_	0/no graph attribute can be smaller than 0	30 for N and LSC (graph attributes counted by number of nodes)/maximum number of nodes for 30 word graphs	Maximum observed value
		29 for RE and ASP (graph attributes counted by number of edges)/maximum number of edges for 30 word graphs)	
*T*	0 for Education/illiterates	30 for education (Post-doctoral level)	12 years of education (High school level)
*f*_*0*_	0/ no graph attribute can be smaller than 0	30 for N and LSC (graph attributes counted by number of nodes)/maximum number of nodes for 30 word graphs	Minimum observed value
		29 for RE and ASP (graph attributes counted by number of edges)/maximum number of edges for 30 word graphs)	

### Code availability

All code is available for scientific purposes by request.

## Electronic supplementary material

Supplemental Figure and Table

## Data Availability

All data are available for scientific purposes by request.

## Electronic supplementary material

**Supplementary information** accompanies the paper on the *npj Schizophrenia* website (10.1038/s41537-018-0067-3).
