# Supplementary material for: The maturation of speech structure in psychosis is resistant to formal education
Source: NPJ Schizophr. 2018 Dec 7;4:25. doi: 10.1038/s41537-018-0067-3 (PMC6286358; doi:10.1038/s41537-018-0067-3)
Supplement: Supplementary file 1 — Supplemental Figure and Table [file 41537_2018_67_MOESM1_ESM.pdf]

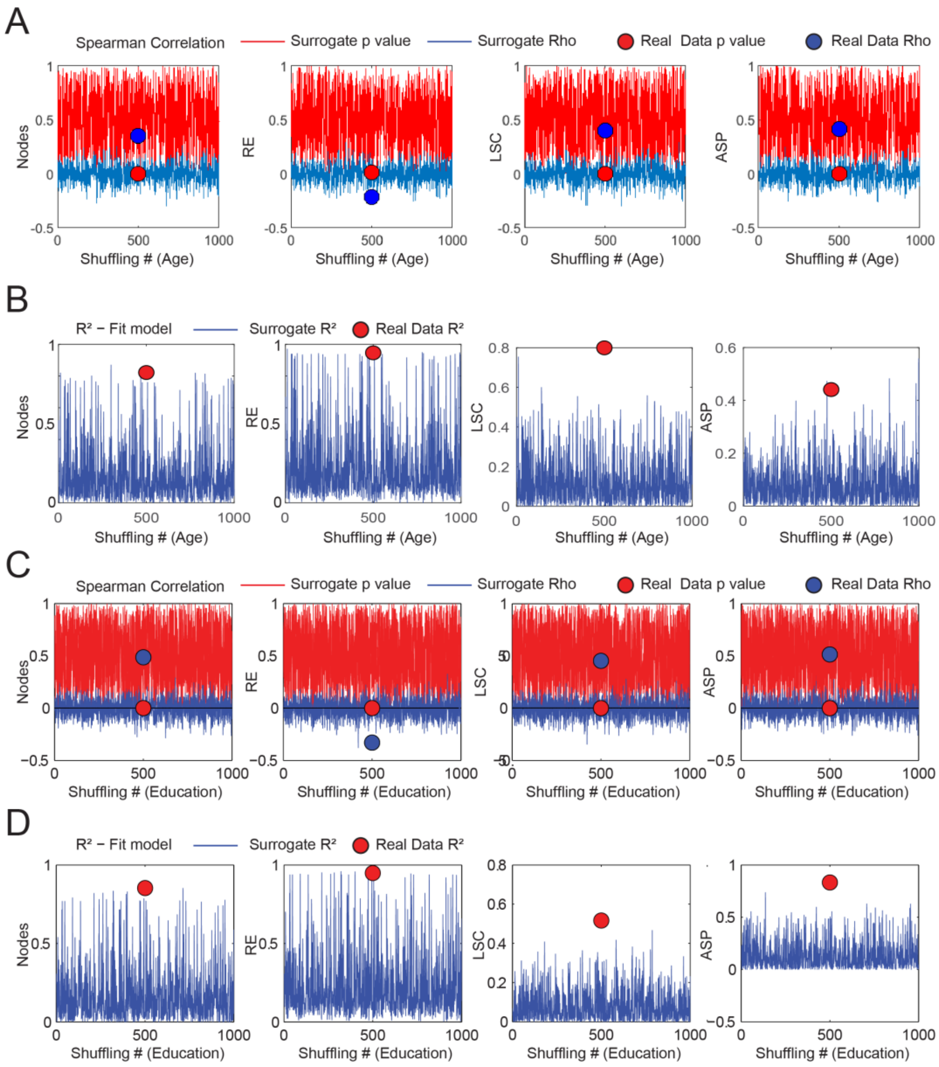

Supplementary Figure S1: Spearman correlation and goodness of fit calculated for surrogated age or educational level. A) Spearman correlations of graph attributes with shuffled or real years of age (lines or dots, respectively). B) Exponential fits of graph attributes with shuffled or real years of age (lines or dots, respectively). C) Spearman correlations of graph attributes with shuffled or real years of education (lines or dots, respectively). D) Exponential fits of graph attributes with shuffled or real years of education (lines or dots, respectively).

Supplementary Table S1: Raw data per subject

Subject ID	Age	School	N	RE	LSC	ASP	RErand	LSCrand	ASPrand	Group
Sub001	2	0	23.00	2.17	10.50	10.00	0.73	3.67	6.89	control
Sub002	3	0	16.33	6.33	5.67	7.00	1.19	5.25	5.32	control
Sub003	3	0	23.00	1.67	16.33	10.33	0.65	3.72	7.26	control
Sub004	3	0	19.00	5.00	1.00	10.00	0.57	3.24	6.70	control
Sub005	5	0	21.29	2.14	13.14	7.43	0.73	3.99	6.62	control
Sub006	30	0	23.80	1.20	13.40	8.80	0.61	3.01	7.51	control
Sub007	41	0	20.29	3.36	13.93	7.93	0.92	4.78	6.21	control
Sub008	5	1	23.00	1.08	14.58	8.83	0.70	3.68	7.06	control
Sub009	6	1	25.00	0.40	11.60	10.80	0.56	2.97	7.82	control
Sub010	6	2	28.67	0.00	1.33	14.00	0.36	2.05	8.25	control
Sub011	6	2	27.00	0.00	2.50	11.50	0.42	2.00	8.27	control
Sub012	6	2	23.00	2.25	13.25	9.25	0.59	3.65	7.09	control
Sub013	6	2	22.25	2.00	14.88	8.38	0.79	4.13	6.72	control
Sub014	6	2	23.43	2.29	13.14	8.86	0.63	3.66	7.20	control
Sub015	7	2	22.50	1.00	8.25	7.50	0.65	3.41	6.91	control
Sub016	7	2	17.00	5.00	11.00	5.00	1.11	5.83	5.37	control
Sub017	7	2	26.40	0.10	16.00	11.20	0.54	3.09	7.99	control
Sub018	7	2	24.80	0.80	7.20	9.60	0.48	2.52	7.80	control
Sub019	7	2	21.22	2.44	16.33	8.11	0.83	4.51	6.45	control
Sub020	7	2	24.50	0.50	2.00	7.50	0.37	1.75	7.66	control
Sub021	7	2	22.00	2.83	11.83	8.00	0.79	4.11	6.79	control
Sub022	7	2	23.38	0.88	10.38	9.38	0.65	3.26	7.32	control
Sub023	7	2	22.57	1.14	14.57	9.00	0.73	3.68	6.95	control
Sub024	7	2	24.60	0.60	13.60	8.80	0.54	2.89	7.70	control
Sub025	7	2	24.13	1.13	15.63	10.50	0.67	3.55	7.37	control
Sub026	7	2	22.00	2.22	15.11	8.11	0.76	4.03	6.81	control
Sub027	7	2	22.86	1.14	15.71	9.29	0.70	3.81	6.97	control
Sub028	7	2	23.00	1.60	15.70	9.40	0.74	4.08	7.04	control
Sub029	7	2	24.00	0.00	7.25	9.75	0.49	2.79	7.67	control
Sub030	7	2	22.20	1.40	15.40	8.40	0.66	3.92	6.97	control
Sub031	7	2	22.86	2.00	13.29	9.43	0.75	3.66	7.23	control
Sub032	7	2	23.00	1.00	3.00	9.00	0.48	2.25	7.45	control
Sub033	7	2	23.43	1.00	13.57	10.14	0.65	3.45	7.15	control
Sub034	7	2	23.50	0.50	7.50	10.75	0.59	3.34	7.40	control
Sub035	7	2	24.67	1.17	12.67	11.50	0.67	3.39	7.47	control
Sub036	7	2	22.38	1.50	15.00	10.00	0.84	4.27	6.87	control
Sub037	7	2	24.80	0.60	8.00	10.20	0.51	2.79	7.73	control
Sub038	7	2	21.00	0.00	7.00	7.00	0.59	2.96	6.78	control
Sub039	7	2	24.33	1.33	3.67	7.33	0.37	2.44	7.71	control
Sub040	7	2	24.50	0.83	9.67	8.67	0.54	3.15	7.54	control
Sub041	7	2	23.33	1.67	13.44	9.89	0.71	3.61	7.03	control
Sub042	7	2	24.67	0.33	8.33	9.67	0.58	2.80	7.70	control
Sub043	7	2	26.33	1.00	6.00	11.33	0.49	2.62	8.08	control
Sub044	7	2	26.00	0.00	6.00	11.00	0.43	2.21	8.02	control
Sub045	7	2	24.20	1.60	14.40	10.10	0.63	3.32	7.44	control
Sub046	7	2	23.11	1.22	13.22	9.11	0.69	3.72	7.12	control

Sub047	7	2	23.13	1.13	15.75	8.75	0.69	3.86	7.04	control
Sub048	7	2	23.83	1.83	11.83	8.50	0.67	3.48	7.34	control
Sub049	7	2	24.67	1.17	6.83	12.67	0.57	2.86	7.64	control
Sub050	7	2	23.50	1.17	7.00	9.17	0.59	3.15	7.46	control
Sub051	7	2	24.50	1.00	9.50	8.50	0.53	2.54	7.70	control
Sub052	7	2	22.83	1.33	16.75	8.75	0.78	4.16	6.92	control
Sub053	7	2	23.00	1.50	9.00	10.33	0.66	3.30	7.26	control
Sub054	7	2	24.60	0.40	8.60	9.60	0.63	3.20	7.66	control
Sub055	7	2	24.25	0.00	8.00	12.00	0.61	3.09	7.55	control
Sub056	8	2	23.00	1.00	13.80	7.00	0.63	3.14	7.35	control
Sub057	8	2	23.57	0.57	14.14	9.71	0.60	3.58	7.39	control
Sub058	8	2	24.40	1.20	9.20	11.80	0.57	3.25	7.57	control
Sub059	8	2	25.50	1.00	2.50	10.00	0.32	2.28	8.07	control
Sub060	8	2	24.14	1.00	12.43	9.57	0.68	3.39	7.39	control
Sub061	8	2	24.17	0.67	14.00	9.17	0.61	3.33	7.47	control
Sub062	8	2	20.67	4.67	8.00	7.00	0.85	4.26	6.44	control
Sub063	8	2	24.00	1.25	7.00	9.00	0.55	2.86	7.51	control
Sub064	8	2	25.00	0.40	11.80	9.40	0.60	3.05	7.67	control
Sub065	8	2	23.00	5.00	1.00	6.00	0.35	2.16	7.54	control
Sub066	8	2	24.83	0.33	18.67	10.17	0.64	3.28	7.60	control
Sub067	8	2	24.20	0.70	15.50	9.50	0.65	3.40	7.44	control
Sub068	8	2	20.75	3.50	8.75	9.00	0.70	3.85	6.57	control
Sub069	8	2	22.50	2.00	5.50	6.75	0.64	2.97	7.10	control
Sub070	8	2	21.88	1.88	13.25	8.13	0.68	3.79	6.82	control
Sub071	8	2	24.00	1.00	6.50	9.75	0.55	2.85	7.58	control
Sub072	8	2	23.00	1.38	12.13	11.38	0.74	3.79	7.12	control
Sub073	8	2	23.50	1.75	12.75	9.25	0.66	3.33	7.32	control
Sub074	8	2	23.80	1.00	16.20	9.40	0.63	3.58	7.13	control
Sub075	8	2	23.63	1.13	16.00	9.25	0.72	3.83	7.15	control
Sub076	8	2	24.00	0.60	14.00	8.80	0.58	3.44	7.44	control
Sub077	8	2	22.67	1.50	14.00	9.83	0.69	3.80	7.00	control
Sub078	8	2	21.00	2.00	4.80	11.20	0.69	3.52	6.84	control
Sub079	8	2	24.00	1.00	3.00	5.00	0.33	1.84	7.27	control
Sub080	8	2	21.00	2.80	9.40	8.20	0.76	3.68	6.73	control
Sub081	8	2	20.67	1.33	6.00	9.33	0.86	3.99	6.40	control
Sub082	8	2	22.60	2.00	11.60	8.00	0.64	3.37	6.97	control
Sub083	23	4.5	25.11	1.11	14.44	11.89	0.62	3.62	7.61	control
Sub084	41	4.5	24.32	0.95	11.77	12.55	0.69	3.56	7.38	control
Sub085	13	6	22.33	2.00	11.00	9.50	0.62	3.60	7.12	control
Sub086	14	6	23.60	1.40	9.60	7.80	0.66	3.46	7.28	control
Sub087	15	6	26.17	0.67	9.00	11.50	0.50	2.96	8.02	control
Sub088	13	7	22.91	1.09	17.00	7.18	0.75	3.94	6.96	control
Sub089	13	7	25.25	0.50	7.25	11.50	0.44	2.54	7.93	control
Sub090	15	7	26.50	1.00	10.50	13.13	0.55	2.97	8.05	control
Sub091	16	7	27.14	0.43	10.57	13.86	0.47	2.67	8.19	control
Sub092	17	7	24.00	1.40	5.40	8.40	0.54	2.76	7.72	control
Sub093	15	8	24.78	1.11	11.78	11.44	0.58	3.13	7.62	control
Sub094	14	9	22.82	1.18	14.91	8.55	0.65	3.67	7.08	control
Sub095	14	9	24.50	1.88	9.50	13.63	0.71	3.79	7.38	control

Sub096	15	9	25.40	0.50	15.30	10.20	0.64	3.12	7.68	control
Sub097	16	9	23.75	1.25	15.13	10.38	0.64	3.32	7.33	control
Sub098	18	9	22.77	1.46	15.40	9.23	0.76	4.16	6.93	control
Sub099	24	10	24.69	0.92	18.54	9.62	0.68	3.66	7.45	control
Sub100	18	12	26.07	0.29	16.00	11.00	0.56	3.24	7.89	control
Sub101	18	12	25.13	0.50	12.25	11.25	0.60	3.05	7.69	control
Sub102	19	12	25.62	0.23	17.62	11.69	0.59	3.33	7.76	control
Sub103	19	12	24.78	0.67	13.67	10.33	0.57	3.32	7.57	control
Sub104	20	12	23.50	0.71	14.36	10.43	0.66	3.36	7.35	control
Sub105	23	12	22.25	1.50	13.13	9.38	0.72	4.21	6.87	control
Sub106	29	12	23.09	1.40	15.66	9.21	0.72	3.78	7.06	control
Sub107	32	12	24.31	1.24	12.59	12.10	0.66	3.46	7.42	control
Sub108	34	12	24.18	1.10	12.92	11.49	0.64	3.31	7.41	control
Sub109	35	12	24.12	1.20	15.20	11.04	0.68	3.66	7.32	control
Sub110	35	12	25.48	0.37	13.74	12.41	0.62	3.20	7.78	control
Sub111	41	12	25.25	0.50	12.13	10.25	0.58	3.13	7.60	control
Sub112	43	12	24.18	1.11	14.18	12.33	0.69	3.74	7.29	control
Sub113	49	12	25.00	0.36	16.36	11.50	0.61	3.29	7.56	control
Sub114	51	12	25.44	0.38	12.81	11.56	0.56	2.90	7.79	control
Sub115	56	12	23.02	1.65	17.31	9.35	0.76	4.07	6.99	control
Sub116	23	14	24.05	0.65	16.65	9.15	0.67	3.45	7.39	control
Sub117	25	15	23.90	0.65	16.55	9.30	0.64	3.43	7.26	control
Sub118	39	16	23.17	1.09	17.26	9.09	0.76	4.05	6.99	control
Sub119	23	17	25.18	0.45	13.91	12.45	0.61	3.30	7.67	control
Sub120	24	17	25.58	0.75	14.25	11.25	0.61	3.42	7.63	control
Sub121	25	17	24.50	1.07	19.36	9.07	0.66	3.56	7.35	control
Sub122	23	18	23.92	1.15	17.38	9.23	0.73	3.71	7.22	control
Sub123	25	18	24.69	0.69	13.56	12.00	0.65	3.42	7.46	control
Sub124	27	18	25.92	0.67	15.92	12.25	0.58	3.16	7.85	control
Sub125	28	18	25.25	0.83	16.58	11.17	0.62	3.39	7.59	control
Sub126	32	18	25.00	1.00	12.20	13.20	0.60	3.28	7.56	control
Sub127	28	20	25.24	0.71	17.76	11.47	0.61	3.30	7.64	control
Sub128	28	20	26.08	0.58	13.92	13.58	0.59	3.13	7.72	control
Sub129	30	20	25.14	0.14	16.36	11.36	0.62	3.35	7.66	control
Sub130	30	20	24.23	1.00	17.31	10.54	0.70	3.73	7.32	control
Sub131	31	20	26.15	0.31	14.77	13.23	0.58	3.20	7.87	control
Sub132	31	20	25.54	0.77	17.08	11.69	0.63	3.29	7.75	control
Sub133	28	23	24.00	1.26	16.74	10.26	0.72	3.86	7.19	control
Sub134	31	23	26.00	0.80	13.90	11.70	0.57	3.18	7.79	control
Sub135	34	23	25.81	1.13	16.69	12.88	0.59	3.19	7.71	control
Sub136	7	2	23.72	1.28	13.86	9.72	0.70	3.64	7.22	psychosis
Sub137	9	3	21.80	2.20	10.40	9.40	0.79	3.89	6.73	psychosis
Sub138	12	4	20.50	2.00	4.00	10.50	0.66	3.13	6.77	psychosis
Sub139	13	0	22.00	3.00	1.00	5.00	0.37	1.71	6.19	psychosis
Sub140	13	4	24.33	0.33	13.00	8.33	0.52	2.75	7.70	psychosis
Sub141	13	7	22.88	2.00	11.88	9.88	0.65	3.44	7.13	psychosis
Sub142	14	9	24.56	1.22	13.22	12.00	0.66	3.53	7.46	psychosis
Sub143	15	6	22.60	1.40	16.33	9.07	0.75	4.17	6.88	psychosis
Sub144	15	8	22.00	3.00	5.00	9.00	0.47	2.56	7.21	psychosis

Sub145	16	0	23.88	0.75	14.25	9.75	0.65	3.34	7.33	psychosis
Sub146	16	6	22.00	2.00	1.00	6.00	0.36	1.61	6.66	psychosis
Sub147	16	7	26.33	0.33	9.67	13.33	0.52	2.97	8.05	psychosis
Sub148	16	7	25.33	0.67	8.33	12.33	0.56	2.81	7.87	psychosis
Sub149	16	8	26.00	0.67	9.33	16.00	0.52	3.08	7.95	psychosis
Sub150	17	2	24.35	0.69	14.54	10.85	0.65	3.45	7.48	psychosis
Sub151	17	4.5	21.75	2.75	15.50	8.25	0.76	3.95	6.91	psychosis
Sub152	17	10	22.42	1.92	14.33	8.25	0.77	4.09	6.90	psychosis
Sub153	18	6	25.75	0.50	12.63	11.50	0.60	3.44	7.79	psychosis
Sub154	18	9	24.00	1.00	9.50	11.25	0.72	3.16	7.48	psychosis
Sub155	18	11	24.18	1.27	13.82	10.82	0.69	3.67	7.31	psychosis
Sub156	21	1	22.89	0.56	1.67	7.11	0.30	1.82	6.86	psychosis
Sub157	22	12	17.00	3.00	1.00	11.00	0.91	3.18	6.03	psychosis
Sub158	23	5	24.86	0.29	6.29	10.00	0.48	2.31	7.68	psychosis
Sub159	23	12	23.05	1.35	8.10	9.25	0.58	3.05	7.31	psychosis
Sub160	23	13	24.67	0.83	8.75	10.92	0.57	3.16	7.65	psychosis
Sub161	24	7	20.67	2.42	7.54	9.04	0.81	3.82	6.62	psychosis
Sub162	24	14	23.50	0.80	16.70	9.10	0.69	3.82	7.17	psychosis
Sub163	25	4.5	23.25	1.00	20.25	8.50	0.80	4.14	7.10	psychosis
Sub164	26	4.5	21.41	2.18	13.24	8.71	0.81	4.06	6.63	psychosis
Sub165	26	12	24.50	0.50	12.13	9.25	0.62	3.25	7.52	psychosis
Sub166	26	12	19.70	2.90	8.20	9.50	0.89	4.28	6.25	psychosis
Sub167	26	12	27.00	0.00	16.00	14.33	0.58	3.40	8.13	psychosis
Sub168	27	4.5	22.83	1.17	18.00	8.67	0.81	4.23	6.87	psychosis
Sub169	28	0	23.15	0.85	9.77	9.08	0.60	3.18	7.23	psychosis
Sub170	28	6	22.96	1.32	14.80	9.76	0.72	3.82	7.09	psychosis
Sub171	29	12	22.50	1.83	13.71	9.71	0.76	4.17	6.86	psychosis
Sub172	30	4.5	26.50	1.00	14.50	15.50	0.65	3.18	7.83	psychosis
Sub173	30	12	27.00	0.25	17.50	14.75	0.58	3.30	7.93	psychosis
Sub174	32	5	22.00	1.29	11.71	6.29	0.56	3.07	7.08	psychosis
Sub175	33	5	21.38	3.28	12.22	10.03	0.88	4.53	6.52	psychosis
Sub176	33	12	24.00	1.24	10.10	10.14	0.59	3.06	7.49	psychosis
Sub177	34	0	23.77	0.71	14.66	9.54	0.65	3.59	7.33	psychosis
Sub178	34	0	23.64	0.96	11.32	9.60	0.65	3.45	7.32	psychosis
Sub179	34	8	23.33	1.00	6.00	7.67	0.44	2.35	7.52	psychosis
Sub180	34	12	23.67	1.11	12.22	11.22	0.60	3.35	7.31	psychosis
Sub181	36	12	23.36	1.36	15.12	9.58	0.70	3.88	7.08	psychosis
Sub182	37	7	23.30	1.05	9.90	10.45	0.61	3.36	7.32	psychosis
Sub183	38	12	22.90	1.40	14.70	9.30	0.69	3.89	6.99	psychosis
Sub184	39	0	23.50	1.50	5.50	10.00	0.48	2.60	7.39	psychosis
Sub185	39	12	23.52	0.52	12.76	10.62	0.65	3.39	7.27	psychosis
Sub186	42	6	23.77	1.05	17.68	9.18	0.72	3.57	7.26	psychosis
Sub187	42	12	23.42	0.84	10.42	9.95	0.64	3.11	7.32	psychosis
Sub188	43	0	22.86	1.86	4.00	12.29	0.59	3.00	7.26	psychosis
Sub189	43	4.5	23.88	0.75	8.13	10.38	0.53	2.71	7.61	psychosis
Sub190	45	16	21.73	1.73	12.80	8.27	0.80	4.09	6.75	psychosis
Sub191	46	12	23.58	1.07	15.51	10.34	0.73	3.92	7.12	psychosis
Sub192	49	0	23.86	0.93	9.00	9.43	0.61	3.18	7.45	psychosis
Sub193	50	4.5	23.29	1.62	11.71	10.33	0.59	3.11	7.26	psychosis

Sub194	51	12	22.25	2.08	9.17	7.67	0.67	3.33	7.09	psychosis
Sub195	51	16	25.60	0.60	19.60	11.60	0.59	3.36	7.51	psychosis
Sub196	51	16	22.25	1.13	14.88	9.75	0.72	3.91	6.82	psychosis
Sub197	53	12	23.40	1.67	15.87	11.00	0.72	3.86	7.12	psychosis
Sub198	57	4.5	21.62	1.92	11.38	9.88	0.78	3.72	6.73	psychosis
Sub199	57	6	24.53	0.80	14.13	9.80	0.55	3.19	7.54	psychosis
Sub200	58	14	22.40	1.91	9.11	9.94	0.69	3.53	7.03	psychosis
Sub201	13	0	24.57	0.57	15.29	12.00	0.60	3.40	7.49	illiterates
Sub202	14	0	20.33	2.00	13.00	8.33	0.83	4.50	6.46	illiterates
Sub203	14	0	25.33	0.67	11.67	11.67	0.53	3.12	7.92	illiterates
Sub204	15	0	28.00	0.00	1.00	9.00	0.40	2.01	7.98	illiterates
Sub205	48	0	23.62	1.31	11.08	12.46	0.74	3.81	7.11	illiterates
Sub206	13	0	26.00	0.75	15.25	11.00	0.57	3.12	7.91	illiterates
Sub207	46	0	24.73	0.91	16.18	10.73	0.58	3.47	7.46	illiterates
Sub208	51	0	25.00	1.00	2.00	9.00	0.47	1.98	7.96	illiterates
Sub209	50	0	23.00	1.36	16.71	8.71	0.79	3.90	7.02	illiterates
Sub210	47	0	23.67	1.00	9.67	13.00	0.71	3.63	7.21	illiterates
Sub211	50	0	21.00	2.00	7.29	8.43	0.81	4.21	6.59	illiterates
Sub212	30	0	23.29	1.14	17.43	9.52	0.73	3.97	7.03	illiterates
Sub213	55	0	24.11	0.89	11.56	11.44	0.58	3.36	7.40	illiterates
Sub214	62	0	21.20	2.10	12.00	10.20	0.86	4.38	6.61	illiterates
Sub215	4	0	23.00	2.33	9.50	9.17	0.73	3.52	7.10	preschool
Sub216	5	0	22.00	2.67	5.33	8.67	0.57	2.69	7.20	preschool
Sub217	3	0	23.25	0.75	10.00	4.50	0.53	2.94	7.37	preschool
Sub218	4	0	21.38	1.88	7.25	8.75	0.80	3.71	6.79	preschool
Sub219	4	0	21.86	1.29	11.14	8.00	0.78	3.83	6.84	preschool
Sub220	3	0	20.67	3.83	11.00	9.00	0.77	4.03	6.53	preschool
Sub221	4	0	24.33	1.00	6.00	10.00	0.58	2.60	7.69	preschool
Sub222	4	0	19.08	3.85	14.46	7.62	1.07	5.36	5.77	preschool
Sub223	5	0	25.00	1.00	6.00	12.00	0.40	2.11	7.80	preschool
Sub224	5	0	23.00	0.56	17.44	7.22	0.74	4.03	6.97	preschool
Sub225	3	0	19.75	2.75	5.50	8.75	0.77	3.70	6.55	preschool
Sub226	4	0	21.00	2.00	2.33	5.33	0.46	2.09	7.00	preschool
Sub227	4	0	21.25	1.50	10.50	6.88	0.79	4.11	6.78	preschool
Sub228	4	0	22.56	1.00	12.78	9.11	0.75	3.99	6.89	preschool
Sub229	2	0	21.50	0.50	2.50	7.00	0.54	2.30	7.36	preschool
Sub230	3	0	20.00	3.80	10.60	8.20	0.81	4.34	6.13	preschool
Sub231	3	0	22.00	0.00	1.00	2.00	0.06	1.08	3.64	preschool
Sub232	4	0	21.25	2.25	4.25	5.75	0.58	2.87	6.84	preschool
